# Immune Checkpoint Inhibitors in Field Cancerization and Keratinocyte Cancer Prevention

**DOI:** 10.1001/jamadermatol.2024.5750

**Published:** 2025-02-12

**Authors:** Charlotte Cox, Susan Brown, Euan Walpole, Edwige Roy, Lea Dousset, Rahul Ladwa, Kiarash Khosrotehrani

**Affiliations:** 1 Dermatology Research Centre, Frazer Institute, Experimental Dermatology Group, University of Queensland, Brisbane, Australia; 2 Department of Dermatology, Princess Alexandra Hospital, Brisbane, Australia; 3 Department of Medical Oncology, Princess Alexandra Hospital, Brisbane, Australia

## Abstract

**Importance:**

Therapies for individual keratinocyte carcinomas (KCs) do not prevent the onset of new KCs in a field of sun damage, and therefore the KC burden remains unchanged.

**Objective:**

To investigate the association of immune checkpoint inhibitors (ICIs) with changes in field cancerization evaluated by the number of actinic keratoses (AKs) and KCs at baseline compared with 12 months after starting ICI therapy.

**Design, Setting, and Participants:**

This prospective cohort study was performed at the outpatient oncology clinic of a single tertiary public hospital in Brisbane, Australia, from April 1, 2022, to November 30, 2023. Consecutive immunocompetent adults starting therapy with an inhibitor for programmed cell death 1 (PD-1) or programmed cell death ligand 1 (PDL-1) for any active cancer, with a planned treatment duration of at least 6 months, and who exhibited clinical AKs on their forearms were eligible. Those with immunosuppression, concurrent chemotherapy or radiotherapy, or recent topical fluorouracil use were excluded.

**Exposures:**

Intravenous ICI therapy, either PD-1 or PDL-1 inhibitors with or without a cytotoxic T-lymphocyte–associated protein 4 inhibitor, with therapy duration determined by the treating oncologist.

**Main Outcomes and Measures:**

Clinical AKs were counted and photographed before and 3, 6, and 12 months after starting ICI therapy. KC numbers were evaluated based on histopathology reports of all skin lesions excised 12 months before and after starting ICI therapy. Participants’ medical history, primary cancer tumor response using Response Evaluation Criteria in Solid Tumors, and adverse events were recorded.

**Results:**

A total of 23 participants were recruited, of whom 17 (73.9%) were male, with a mean (SD) age of 69.7 (9.6) years. No participants withdrew; however, 4 died during the study due to disease progression. The mean (SD) AK number significantly decreased from 47.2 (33.8) at baseline to 14.3 (12.0) at 12 months (*P* < .001). Younger patients (8 of 12 [66.7%] vs 4 of 12 [33.3%]; *P* = .007) and those with a history of blistering sunburn (12 of 12 [100%] vs 0; *P* = .005) were more likely to reduce their AK numbers by 65% or greater. KC total numbers decreased from 42 in the 12 months before starting ICI therapy to 17 in the 12 months after. The number of cutaneous squamous cell carcinomas decreased from 16 to 5 in the same period.

**Conclusions and Relevance:**

This pilot cohort study found that ICIs used for any cancer were associated with a significant reduction of AKs, suggesting potential as an immunopreventive strategy for high-risk individuals. Given the known effects of other chemopreventive agents on KCs, further investigation into ICIs managing field cancerization is required, especially considering toxicity and cost.

## Key Points

**Question:**

How are immune checkpoint inhibitors (ICIs) associated with changes in field cancerization and keratinocyte cancer occurrence?

**Findings:**

In this cohort study of 23 adults starting ICI therapy for any cancer, the mean number of actinic keratoses on the forearms significantly decreased from 47 at baseline to 14 at 12 months after therapy initiation. Younger patients and those with a history of blistering sunburn were more likely to reduce their actinic keratosis numbers by 65% or greater.

**Meaning:**

These results suggest that ICIs may provide benefit for field cancerization as an immunopreventive strategy in high-risk populations.

## Teaser

This cohort study investigates the association of immune checkpoint inhibitor therapy with changes in field cancerization and the development of new keratinocyte carcinoma during a 1-year follow-up in patients with any cancer.

## Introduction

Keratinocyte carcinomas (KCs) are the most common cancer diagnosed in humans each year and include basal cell carcinoma (BCC) and cutaneous squamous cell carcinoma (cSCC).^1^ Actinic keratoses (AKs) are discrete scaly focal areas of dysplasia and are both a risk factor for and precursor to cSCC.^2^ The estimated risk of developing cSCC from a preexisting AK is approximately 0.01% to 0.025% per lesion-year. However, this risk increases to 16% to 20% when a patient has more than 20 AKs, reflecting field cancerization.^3,4,5,6^ Field cancerization is defined as an area of skin chronically exposed to UV radiation, resulting in subclinical mutations found in most cSCCs, leading to both AKs and multiple KCs over time.^7,8^ Current therapies for individual KCs are suboptimal at reducing their significant burden, as they do not prevent the onset of new KCs within an area of field cancerization. Therefore, field-based therapy rather than lesion-based therapy is required to manage the disease burden.^9^

Immune checkpoint inhibitors (ICIs) constitute an immunotherapy approved to treat a range of advanced cancers, including KCs.^10,11,12^ Survival outcomes have dramatically improved with immunotherapy, and objective response rates occur between 44% to 60% with cemiplimab and 34% with pembrolizumab for patients with advanced cSCC.^13,14,15,16,17^ However, the potential of ICIs as systemic field therapy to prevent new KCs in patients at high risk is unknown. ICIs are most effective on tumors with a high tumor mutation burden. KCs, along with clinically normal photoexposed skin, have the highest tumor mutation burden of all cancers.^7,8^

We aimed to investigate the effect of ICI therapy on field cancerization and the development of new KCs during a 1-year follow-up. We hypothesized that ICI therapy would result in a reduction of the incidence in AKs and KCs. We prospectively assessed clinical AK evolution and the development of new KCs, including cSCC and BCC, that required excision in a cohort of patients starting ICI therapy for any cancer.

## Methods

### Study Design

This prospective, single-center, cohort study recruited and followed up patients from the outpatient oncology clinic of a single tertiary public hospital in Brisbane, Australia, from April 1, 2022, to November 30, 2023. Ethics approval was obtained from the Metro South Human Research Ethics Committee, and written informed consent was acquired from all participants. We followed the Strengthening the Reporting of Observational Studies in Epidemiology (STROBE) reporting guideline for cohort studies. Our primary study outcome was to determine the effect of intravenous ICI on the number of AKs at 12 months after starting ICI therapy, and the secondary outcome was the number of KCs excised in the 12-month period preceding and following the start of intravenous ICI therapy.

Immunocompetent adults starting therapy with either an inhibitor for programmed cell death 1 (PD-1) or programmed cell death ligand 1 (PDL-1), with or without a cytotoxic T-lymphocyte–associated protein 4 inhibitor, for any active cancer and with a minimum planned treatment duration of 6 months were approached and further eligible if they exhibited clinical field cancerization (defined as a minimum of 6 AKs) on their bilateral forearms (as a representation of photodamaged skin). Participants were excluded if they were immunocompromised (with immunodeficiency, hematological disorders, or use of immunosuppressive drugs), receiving concurrent radiotherapy or chemotherapy with fluorouracil or its precursors, had a genetic condition predisposing them to skin cancer, were taking nicotinamide, or had used field therapy such as topical fluorouracil, cryotherapy, or photodynamic therapy within the past 6 months. These factors were excluded to minimize potential sources of bias.

After recruitment and obtaining informed written consent, the number of clinical AKs were counted and photographed by a dermatologist (C.C. or K.K.) before initiating ICI therapy and then at 3, 6, and 12 months after starting treatment. No field therapy or chemoprevention was used during the 12-month follow-up period, and this was verified at every visit. Each patient was monitored in our clinic specifically to ensure that the effects of ICI therapy on their skin were accurately assessed. Patients had regular skin checks and reported any interventions by their practitioners. At baseline, participants’ medical history, including skin cancer risk factors and history, was recorded. The primary cancer tumor response to ICI at 3, 6, and 12 months was assessed according to Response Evaluation Criteria in Solid Tumors guidelines, version 1.1.^18^ At each review, any immune-related adverse events were recorded. Additionally, histopathology reports of all skin lesions from the entire body excised in the 12 months before and after starting ICI therapy were collected from electronic medical records and private laboratories. All potential KCs were biopsied and excised as appropriate, and histological confirmation was obtained.

### Statistical Analysis

Target recruitment was determined assuming a normal distribution of data and an SD twice the frequency of AK counts observed in a previous study.^19^ Therefore, 21 patients were needed to reach a significance level of .05 with a power of 80% to observe a 20% decline. Analysis was conducted using SPSS Statistics software, version 28 (IBM Corporation). Paired *t* tests were used to compare the change in the number of AKs at baseline with those at 3, 6, and 12 months. To determine whether specific patient characteristics were associated with AK reduction, we used the 12-month mean clinical AK clearance rate as a threshold: that is, individuals with a clearance rate at or above the mean or below the mean, which was 62.4% and rounded to 65%. To address missing data points, we chose to prospectively follow up patients either until the completion of the 12-month period or their last follow-up, acknowledging not all patients may remain alive or be available for follow-up at 12 months due to their advanced solid cancers. The association between variables was assessed using cross-tabulations with Pearson χ^2^ test. Frequencies and percentages were used to describe categorical variables, while means and SDs were calculated for continuous variables. The significance threshold was set at 2-tailed *P* = .05. We used Prism, version 10.1.2 (GraphPad), to generate graphs.

## Results

### Recruitment

**Figure 1.**
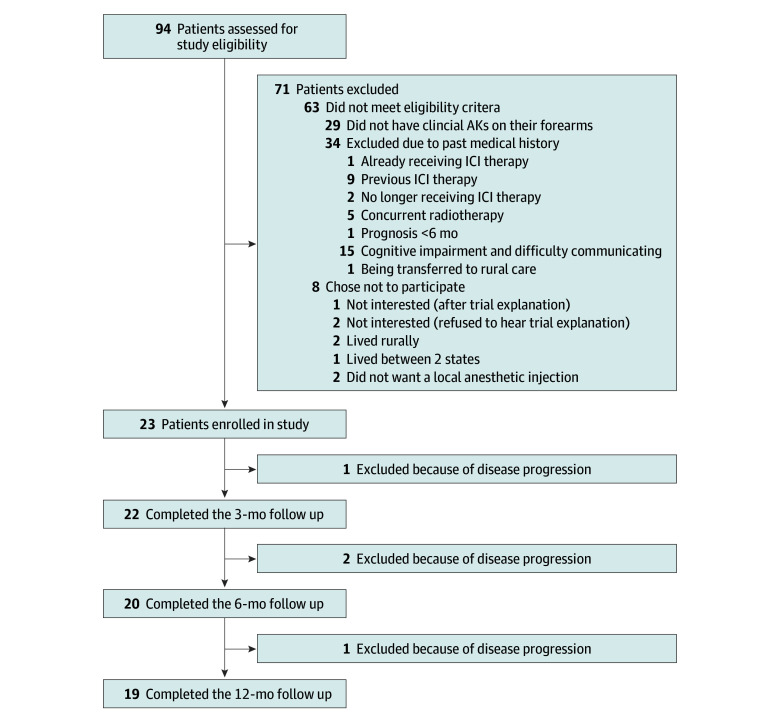
Study Flow Diagram AKs indicates actinic keratoses; ICI, immune checkpoint inhibitor.

Of 94 potential participants who were newly prescribed ICI and approached, 23 were recruited, 63 did not meet the eligibility criteria, and 8 chose not to participate (Figure 1). Most individuals not eligible lacked field cancerization and AKs on forearms. Nineteen participants successfully completed the 12-month review period. Although no participants voluntarily withdrew, 4 died during the study: 1 before the 3-month review, 2 before the 6-month review, and 1 before the 12-month review.

### Patient Characteristics

The demographic characteristics of the 23 recruited participants are presented in Table 1. Among these participants, 6 (26.1%) were female and 17 (73.9%) were male, with a mean (SD) age of 69.7 (9.6) years. Additionally, 15 participants (65.2%) were prescribed monotherapy with an anti–PD-1 antibody, while 8 (34.8%) received a combination of anti–PD-1 and anti–cytotoxic T-lymphocyte–associated protein 4 antibodies. The most frequently prescribed ICI regimen was combination nivolumab and ipilimumab (8 [34.8%]), followed by nivolumab monotherapy (6 [26.1%]), and cemiplimab (5 [21.7%]) or pembrolizumab (4 [17.4%]) monotherapy. More than half of the patients received ICI therapy for skin cancer, with 7 (30.4%) having melanoma and 6 (26.1%) having cSCC. This was followed by 8 patients (34.8%) with lung cancer, and 2 additional cases of tonsillar and renal cell carcinomas. Five of the 23 patients did not receive their planned 6-month infusion of ICI, primarily due to toxic effects or disease progression. Focusing specifically on their skin cancer risk factors, 16 patients (69.6%) had a history of skin cancer, 20 (87.0%) had a fair skin phototype (Fitzpatrick skin type I or II), 17 (73.9%) had a history of blistering sunburns, and 16 (69.6%) were born in Queensland, Australia. Among the 19 patients who completed the study, 7 (36.8%) stopped ICI therapy prior to the 12-month visit, with a mean (SD) time from cessation of 6.3 (1.7) months.

**Table 1.** Patient Characteristics

Characteristic	No. (%) of patients (N = 23)
Sex	
Male	17 (73.9)
Female	6 (26.1)
Age, mean (SD), y	69.7 (9.6)
Fitzpatrick skin type	
I	9 (39.1)
II	11 (47.8)
III	3 (13.0)
History of skin cancer	
Yes	16 (69.6)
No	7 (30.4)
Born in Queensland, Australia	
Yes	16 (69.6)
No	7 (30.4)
History of blistering sunburns	
Yes	17 (73.9)
No	6 (26.1)
Cancer type	
Skin	13 (56.5)
Not skin	10 (43.5)
ICI therapy	
Anti–PD-1	15 (65.2)
Anti–PD-1 with or without anti–CTLA-4	8 (34.8)

Abbreviations: CTLA-4, cytotoxic T-lymphocyte–associated protein 4; ICI, immune checkpoint inhibitor; PD-1, programmed cell death 1 inhibitor.

### Number of Clinical AKs

**Figure 2.**
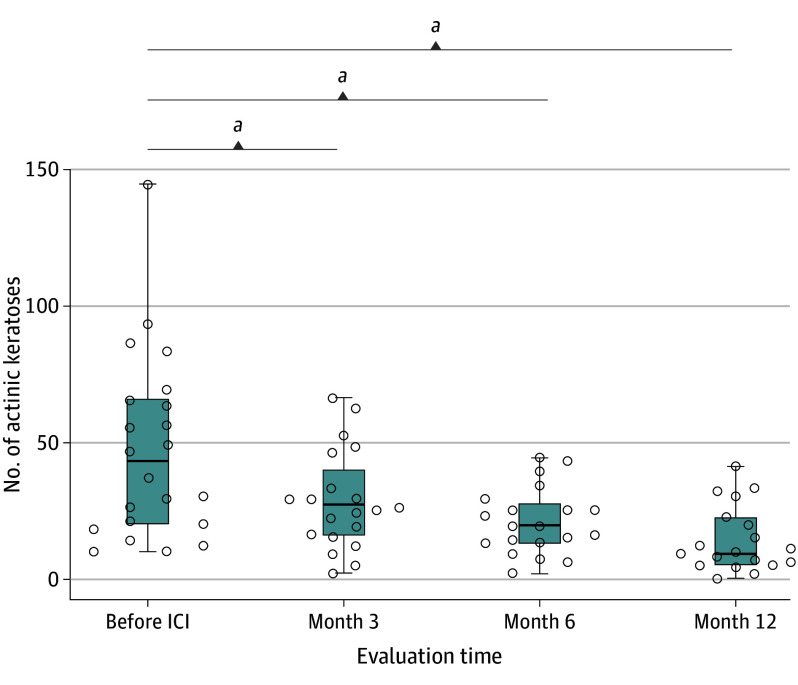
Number of Clinical Actinic Keratoses at Each Time Point After Starting Immune Checkpoint Inhibitor (ICI) Therapy Circles represent individual patients; midlines, means; and whiskers, SDs. ^a^*P* < .001, paired *t* test.

**Figure 3.**
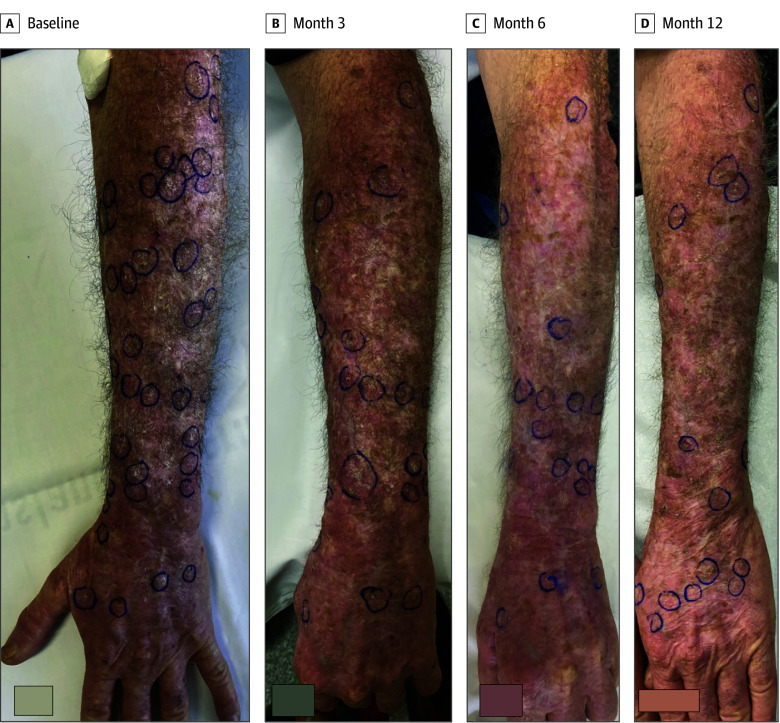
Reduction in Actinic Keratoses Count After 12 Months of Immune Checkpoint Inhibitor Therapy Baseline indicates before the first infusion of immune checkpoint inhibitors. Boxes at the bottom of panels were added to cover text.

Compared with baseline, the AK count decreased in 18 of 22 participants (81.8%) at 3 months and in all participants by 12 months or their final evaluation. The mean (SD) count decreased from 47.2 (33.8) at baseline to 28.9 (17.4; *P* < .001) at 3 months, 21.1 (12.1; *P* < .001) at 6 months, and 14.3 (12.0; *P* < .001) at 12 months, as shown in Figure 2 and Table 2. The mean (SD) clinical AK clearance rate at 12 months was 62.4% (19.3%). Among the patients, the clinical AK count in 1 individual decreased by less than 30%; in 9, by 30% to 64%, and in 12, by more than 65% or more. These included 5 individuals whose clinical AK count reduced by more than 80%. One individual achieved complete resolution. Figure 3 shows a participant with a more than 65% reduction in clinical AK count. Among the 7 patients who discontinued ICI therapy, 4 continued to show a decrease in the number of AKs, while the other 3 experienced regrowth of lesions (eTable 1 in Supplement 1).

**Table 2.** Participant Cancer Type, Treatment, Treatment Duration, and Changes in Clinical AKs From Baseline to 12 Months After ICI Infusion

Cancer type	Type of ICI	Time receiving ICI, mo	No. of clinical AKs			AK change at month 12 or final time point, %	KC count		Immune-related adverse events	
			At baseline	At month 6	At month 12					
							12 mo Before ICI	12 mo After ICI	Cutaneous	Other
cSCC	Cemiplimab	12	30	16	5	−83.4	11	6	None	None
cSCC	Cemiplimab	12	10	29	6	−40.0	1	0	None	None
cSCC	Nivolumab	12	93	39	32	−65.6	3	8	None	None
cSCC	Cemiplimab	8	83	23	41	−50.6	0	2	Rash (BP)	None
cSCC	Cemiplimab	12	69	25	23	−66.7	1	0	None	None
cSCC	Cemiplimab	4	56	19	30	−46.4	3	0	None	Fatigue
Melanoma	Ipilimumab-nivolumab	12	21	14	9	−57.1	0	0	Rash	Chronic cough
Melanoma	Nivolumab	5	55	Deceased	NA	−30.9	0	0^a^	None	None
Melanoma	Ipilimumab-nivolumab	6	10	6	2	−80.0	0	0	Rash	None
Melanoma	Ipilimumab-nivolumab	7	39	25	7	−82.1	0	0	Rash	Pneumonitis, hepatitis
Melanoma	Nivolumab	12	86	43	33	−61.6	1	0	None	None
Melanoma	Ipilimumab-nivolumab	2	12	2	4	−66.7	0	0	None	Colitis, myasthenia
Melanoma	Nivolumab	12	49	20	11	−77.6	1	0	None	None
Lung	Pembrolizumab	<1	7	Deceased	NA	NA	0	0^a^	None	None
Lung	Ipilimumab-nivolumab	7	14	9	8	−42.9	1	0	Rash	None
Lung	Ipilimumab-nivolumab	5	47	34	20	−57.4	0	0	Rash	Xerostomia
Lung	Pembrolizumab	12	18	13	15	−16.7	0	1	None	Nephritis
Lung	Ipilimumab-nivolumab	10	144	44	Deceased	−69.4	20	0^a^	Rash	Thyroiditis, arthralgia, xerostomia
Lung	Pembrolizumab	12	20	7	5	−75.0	0	0	None	None
Lung	Pembrolizumab	12	65	15	0	−100.0	0	0	None	Arthralgia
Lung	Ipilimumab-nivolumab	5	26	Deceased	NA	−53.8	0	0^a^	None	None
Tonsillar	Nivolumab	12	29	13	9	−69.0	0	0	None	None
RCC	Nivolumab	12	63	25	12	−81.0	0	0	None	None

Abbreviations: AKs, actinic keratoses; BP, bullous pemphigoid; ICI, immune checkpoint inhibitor; KC, keratinocyte carcinoma; NA, not available or applicable; RCC, renal cell carinoma; cSCC, cutaneous squamous cell carcinoma.

^a^ Duration of follow-up was reduced and matched before and after ICI.

### Clinical Characteristics and Reduction in AKs

Based on the mean clinical AK clearance rate at 12 months (62.4%), a threshold of 65% was used to divide individuals with clearance rates of 65% or greater and less than 65%. The association between specific patient characteristics and the subsequent reduction in clinical AKs is summarized in eTable 2 in Supplement 1. Individuals younger than 65 years were more likely to have a clearance of 65% or greater (8 of 12 [66.7%] vs 4 of 12 [33.3%]; *P* = .007). Individuals with a history of blistering sunburns were statistically more likely to have experienced a decrease in AK count of 65% or greater compared with those with no history of blistering sunburn (12 of 12 [100%] vs 0; *P* = .005). Interestingly, the mean (SD) clinical AK count for individuals with vs without a history of blistering sunburns was comparable (47.2 [35.2] vs 45.3 [16.7]) at baseline. As expected, individuals with a Fitzpatrick skin type I or II were statistically more likely to have a history of blistering sunburn (9 of 9 or 7 of 10 vs 1 of 3; *P* = .04). There was no association between the clearance rate and the phototype (clearance rate ≥65% in 5 of 9 patients with type I, 6 of 10 with type II, and 1 of 3 with type III; *P* = .72).

### Tumor Response Rate to ICI Therapy and AK Clearance

For the response to ICI therapy, 3 patients were excluded due to rapid disease progression. All patients with metastatic melanoma or cSCC responded to ICI therapy at 3 and 12 months, with 5 of 10 achieving a complete response at 12 months and no longer requiring intravenous ICI treatment (eTable 3 in Supplement 1). Two patients with melanoma received adjuvant ICI treatment and had no recurrence after 1 year of treatment and follow-up. Primary cancer response was not associated with AK clearance of 65% or more upon ICI therapy (8 of 8 [100%] for <65% vs 9 of 12 [75.0%] for ≥65%; *P* = .09) (eTable 2 in Supplement 1).

### Adverse Events With ICI Therapy and AK Clearance

Adverse events occurred in 11 participants (47.8%) as summarized in Table 2. The most common adverse event was a maculopapular rash or pruritus, with 7 participants (30.4%) experiencing a cutaneous skin reaction. One participant developed bullous pemphigoid, requiring oral prednisolone, topical betamethasone, and a 2-week break from ICI infusions. Adverse events in 4 of 22 cases (18.2%) resulted in a treatment break or change in therapy. The adverse events in these 4 participants included nephritis, bullous pemphigoid, and concurrent colitis and myasthenia in 1 participant each and concurrent pneumonitis and hepatitis in 1 participant. Some participants experienced an increase in inflammation (redness and change in palpation) of their AKs at 3 months (eFigure in Supplement 1). There was no difference in the clearance rate of AKs (≥65% or <65%) in those with cutaneous (2 of 4 patients) vs noncutaneous (3 of 7 patients) adverse events (*P* = .82).

### Occurrence of KCs Before and After 12 Months of ICI Therapy

Excised and histologically confirmed KCs from the entire body were counted and compared in the 12 months prior to and after the initiation of ICI therapy in the 19 patients who completed their 12-month follow-up visit, as seen in Table 2. For the 4 individuals deceased during the study, the period before was matched to the period they survived after ICI initiation. The total number of KCs before and after 12 months of ICI therapy decreased from 42 to 17, as summarized in eTable 4 in Supplement 1. Seven participants had KCs occur in the 12 months preceding or following the start of ICI therapy. The mean (SD) number of KCs per individual decreased from 1.91 (0.89) to 0.77 (0.45) (*P* = .26). When assessing individual KC subtypes, the mean (SD) number of cSCCs decreased from 0.73 (0.35) to 0.23 (0.16), representing an absolute reduction from 16 to 5 cSCCs without reaching significance (*P* = .15). The mean (SD) number of BCCs decreased from 0.27 (0.70) to 0.14 (0.35), and the mean (SD) number of intraepidermal carcinomas decreased from 0.86 (2.99) to 0.36 (1.22), though neither reduction reached statistical significance (the absolute number of BCCs decreased from 6 to 3, and the absolute number of intraepidermal carcinomas decreased from 19 to 8).

## Discussion

KCs, specifically BCC and SCC of the skin, are the most common malignant neoplasms in humans. They often occur on photodamaged skin due to UV exposure. Although individual lesions can be easily treated with surgical excision, this does not prevent the onset of new lesions. The rate of new incident lesions in the same area of field cancerization is high, contributing significantly to the overall burden of the disease.^9^ Consequently, strategies are needed that can reduce the onset of new KCs in sun-damaged areas. Our study found that ICI therapy, started for any cancer, led to a significant reduction in the incidence of clinical AKs at 3, 6, and 12 months after starting therapy on bilateral forearms. Moreover, we observed that the beneficial effect was not confined to the period during ICI therapy but may also extend beyond its cessation in some cases. However, the reduction in the number of KCs and particularly SCCs in the 12 months following the start of ICI therapy compared with the 12 months prior was not statistically significant. These findings suggest possible benefits of ICI therapy on field cancerization and the occurrence of cSCC.

Our study explored the association of ICI therapy with changes in field cancerization by assessing the number of clinical AKs, a known precursor to cSCC. Additionally, individuals with a high number of AKs are known to be at a greater risk of having a higher burden of KCs.^7^ Current therapies for field cancerization, such as topical fluorouracil, imiquimod, and photodynamic therapy, are localized and target specific areas.^6,20^ In a recent head-to-head comparison study,^21^ topical fluorouracil was identified as the most effective topical therapy, reducing AKs by more than 75% at 12 months in 75% of patients. This is more effective than the reduction reported herein with ICIs. Similarly, 12 months after treatment, a course of topical fluorouracil was associated with a 75% reduction in cSCCs^21^ compared with a nonsignificant 69% reduction in our study. Systemic chemopreventive agents such as nicotinamide, acitretin, and capecitabine offer another approach. Acitretin has shown a reduction in cSCCs by about 60% in high-risk individuals.^22^ In comparison, nicotinamide has been found to reduce AKs and KCs by 11% and 23%, respectively, at 12 months.^23^ Finally, the chemotherapeutic drug capecitabine has been used off-label at a low dosage to prevent development of cSCC. Although capecitabine may be associated with a reduction in cSCC in high-risk individuals, to our knowledge, only case studies and series have explored capecitabine use.^24^ Although direct comparison of these preexisting chemopreventive agents to our study is challenging, the immunopreventive effect of ICIs appears comparable to those of the best currently available therapies. However, the adverse events associated with ICIs are likely more severe.

A key challenge with ICI therapy is better defining which high-risk patients could benefit the most, especially given the high costs of these drugs.^25^ A recent study^7^ defined a high burden of KCs as more than 10 KCs in the past 5 years and a low burden as less than 3 KCs in the past 5 years. As this was a pilot study, the participants in our study had a range of skin cancer history, and the main objective was to explore the association with clinical AKs. The use of ICI therapy for systemic field cancerization in individuals with specifically a high burden of KC warrants further exploration in future larger studies.

In our study, few predictive patient factors were associated with a reduction of 65% or greater in clinical AKs on bilateral forearms. However, individuals with a history of blistering sunburns were statistically more likely to experience a decrease in clinical AK count of 65% or greater. UV radiation is considered the most influential environmental risk factor for both BCC and cSCC, and a history of blistering sunburns is associated with both types.^26^ Additionally, individuals with a Fitzpatrick skin type I or II were statistically more likely to have a history of blistering sunburns. A low Fitzpatrick skin type is a known risk factor for sunburn and has been linked to increased mutational burden in sun-damaged epidermis.^5,7^ Similarly, older age is associated with a larger mutant clone size and a greater reduction in AKs. Thus, individuals with a low Fitzpatric skin type and history of blistering sunburns may harbor more mutations, potentially increasing their immune targets for ICI therapy. ICIs have demonstrated enhanced efficacy in settings with higher tumor mutational burden, such as metastatic melanoma.^8,27^ Overall, predicting which patients will experience the greatest reduction in clinical AK count based on specific characteristics remains challenging; however, this warrants further exploration in future larger studies.

Although ICIs are relatively well tolerated, adverse events are common and often permanent.^28^ Studies indicate that 30% to 60% of patients develop a cutaneous adverse event, which is the type most frequently reported.^29,30^ This aligns with our findings, where there were no new safety signals and the most prevalent adverse event was a cutaneous skin reaction. Importantly, we observed that immune-related adverse events were not associated with greater reduction in clinical AK count.

### Strengths and Limitations

A major strength of this study was its prospective design. Most potential participants were eligible, and most of those eligible and approached consented to participate in the study. Additionally, there were no withdrawals. A limitation was the assessment of photodamaged skin; currently, counting clinical AKs is the best available measure, as previously described and used.^31^ However, interrater reliability issues occur when counting AKs, and the accuracy of counting AKs can be impacted as some AKs coalesce. In addition, AKs can spontaneously regress. Another limitation was that the 12-month follow-up period was not completed for all patients (4 of the 23 recruited). The observations around the change in the number of AKs following cessation of ICIs therapy are limited in this study; therefore, in future studies it would be beneficial to continue monitoring patients for a minimum of 12 months after cessation of ICI therapy. The assessment of KCs was retrospective and based on histopathology reports rather than systematic skin examination. Moreover, there was heterogeneity in KC occurrence among patients, as 1 patient had a higher rate of KCs, potentially impacting results. In addition, surveillance bias may impact the number of KCs occurring in the 12-month period following ICI therapy initiation, as clinicians may delay procedures while patients are undergoing active cancer treatment or entering end of life or palliative care. Some participant factors may also have been influenced by recall bias.

## Conclusions

This pilot cohort study highlights the potential association of ICI therapy, originally used in cancer treatment, with significant reduction of clinical AKs. However, the observed reduction in cSCCs occurrence was not statistically significant. These findings underscore ICIs’ potential as a novel approach to mitigating field cancerization in high-risk populations. Given the potentially life-threatening adverse events associated with ICI therapy, this pilot study could pave the way for future prospective clinical trials to explore immunopreventive strategies in individuals at extreme risk of KCs when alternative strategies are not viable.

## Notes

Supplement 1. **eFigure.** Cancerization Field on the Forearm **eTable 1.** Characteristics of Participants Who Stopped Immune Checkpoint Inhibitor Therapy and Trends in AK **eTable 2.** Comparison of Individual Characteristics Between Participants With a Decrease in Clinical Actinic Keratoses Count of Less Than 65% Compared With 65% or Greater **eTable 3.** Participant Cancer Type, Treatment, Treatment Response, and Changes in Clinical AKs From Baseline to 12 Months After Immune Checkpoint Inhibitor Infusion **eTable 4.** The Number of KCs in the Year Before and After Starting ICI Therapy

Supplement 2.
Data Sharing Statement
