# Supplementary material for: Immune Checkpoint Inhibitors in Field Cancerization and Keratinocyte Cancer Prevention
Source: JAMA Dermatol. 2025 Feb 12;161(4):383–90. doi: 10.1001/jamadermatol.2024.5750 (PMC11822595; doi:10.1001/jamadermatol.2024.5750)
Supplement: Supplement 1. — eFigure. Cancerization Field on the Forearm eTable 1. Characteristics of Participants Who Stopped Immune Checkpoint Inhibitor Therapy and Trends in AK eTable 2. Comparison of Individual Characteristics Between Participants With a Decrease in Clinical Actinic Keratoses Count of Less Than 65% Compared With 65% or Greater eTable 3. Participant Cancer Type, Treatment, Treatment Response, and Changes in Clinical AKs From Baseline to 12 Months After Immune Checkpoint Inhibitor Infusion eTable 4. The Number of KCs in the Year Before and After Starting ICI Therapy [file jamadermatol-e245750-s001.pdf]

Supplementary Online Content

Cox C, Brown S, Walpole E, et al. Association of immune checkpoint inhibitors with field cancerization and keratinocyte cancer prevention. *JAMA Dermatol*. Published online February 12, 2025. doi:10.1001/jamadermatol.2024.5750

eFigure. Cancerization Field on the Forearm

eTable 1. Characteristics of Participants Who Stopped Immune Checkpoint Inhibitor Therapy and Trends in AK

eTable 2. Comparison of Individual Characteristics Between Participants With a Decrease in Clinical Actinic Keratoses Count of Less Than 65% Compared With 65% or Greater

eTable 3. Participant Cancer Type, Treatment, Treatment Response, and Changes in Clinical AKs From Baseline to 12 Months After Immune Checkpoint Inhibitor Infusion

eTable 4. The Number of KCs in the Year Before and After Starting ICI Therapy

This supplementary material has been provided by the authors to give readers additional information about their work.

© 2025 Cox C, et al. *JAMA Dermatology*.

eFigure. Cancerization Field on the Forearm

Before (A) and after 3 months of PD-1 inhibitors showing inflamed actinic keratoses (B).

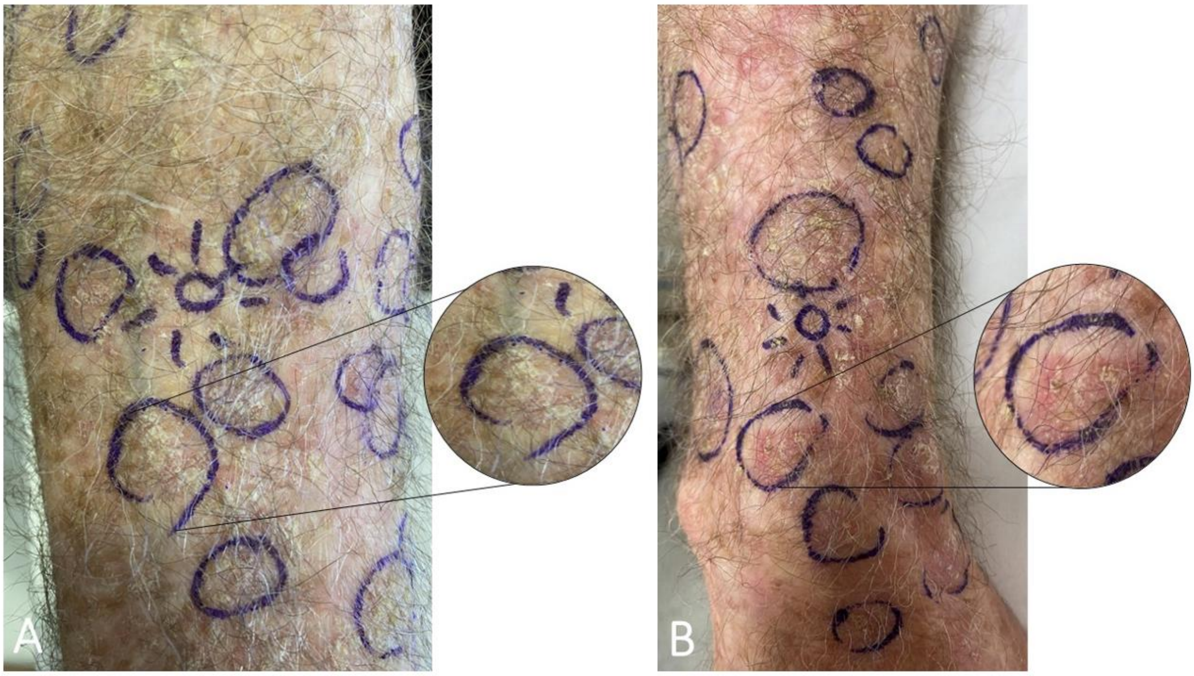

© 2025 Cox C, et al. *JAMA Dermatology*.

eTable 1. Characteristics of Participants Who Stopped Immune Checkpoint Inhibitor Therapy and Trends in AK

Primary Cancer	ICI	No. Months on ICI	No. months after cessation of ICI	Clinical AK Count			% AK change from M0 to M12	% AK change M6 to M12
				M0	M6	M12		
cSCC	Cemip	8	4	83	23	41	-50.6	56.1
cSCC	Cemip	4	8	56	19	30	-46.4	63
Melanoma	Ipi/Nivo	6	6	10	6	2	-80.0	-66.8
Melanoma	Ipi/Nivo	7	5	39	25	7	-82.1	-72
Melanoma	Ipi/Nivo	3	9	12	2	4	-66.7	50
Lung	Ipi/Nivo	7	5	14	9	8	-42.9	-11
Lung	Ipi/Nivo	5	7	47	34	20	-57.4	-41.2

AK = actinic keratoses; Cemip = Cemiplimab; cSCC = cutaneous squamous cell carcinoma; ICI = Immune checkpoint inhibitor; Ipi/Nivo = combination Ipilimumab and Nivolumab therapy; M0 = Month 0; M6 = Month 6; M12 = Month 12; No. = number

© 2025 Cox C, et al. *JAMA Dermatology*.

eTable 2. Comparison of Individual Characteristics Between Participants With a Decrease in Clinical Actinic Keratoses Count of Less Than 65% Compared With 65% or Greater

	**Individual clinical actinic keratoses trend**		
	**Decrease by <65% (N=10)**	**Decrease by ≥65% (N=12)**	**p-value***
**Sex**			
Male	7 (70)	9 (75)	0.79
Female	3 (30)	3 (25)	
**Age**			
<65 years old	1 (10)	8 (67)	**0.007**
≥65 years old	9 (90)	4 (33)	
**Type of Primary Cancer**			
cSCC	3 (30)	3 (25)	0.54
Melanoma	3 (30)	4 (33)	
Lung	4 (40)	3 (25)	
Other	0	2 (17)	
**Primary cancer origin**			
Skin cancer	6 (60)	7 (58)	0.94
Other cancer	4 (40)	5 (42)	
**Primary cancer metastasised**			
Yes	5 (50)	7 (58)	0.70
No	5 (50)	5 (42)	
**Type of ICI**			
Nivolumab	2 (20)	4 (33)	0.80
Nivolumab / Ipilimumab	4 (40)	4 (33)	
Cemiplimab	3 (30)	2 (17)	
Pembrolizumab	1 (10)	2 (17)	
**ICI Monotherapy vs Combination**			
Anti-PD1 antibody	6 (60)	8 (67)	0.75
Anti-PD1 & anti-CTLA4 antibodies	4 (40)	4 (33)	
**Primary cancer responded to ICI therapy**			
Yes**	8 (100)	9 (75)	0.086
No	0	3 (25)	
**ICI therapy side effects**			
Yes	6 (60)	5 (42)	0.39
No	4 (40)	7 (58)	
**Fitzpatrick skin type**			
Type I	4 (40)	5 (42)	0.72
Type II	4 (40)	6 (50)	
Type III	2 (20)	1 (8)	
**Born in Queensland**			
Yes	6 (60)	10 (83)	0.22
No	4 (40)	2 (17)	
**History of blistering sunburn**			
Yes	5 (50)	12 (100)	**0.005**
No	5 (50)	0	
**History of skin cancer**			
Yes	6 (60)	10 (83)	0.22
No	4 (40)	2 (17)	

*Values are N (%). *Chi-square P-value; **Only 20/22 patients could be evaluated for therapy response and AK count. cSCC = Cutaneous Squamous Cell Carcinoma; ICI = immune checkpoint inhibitor.*

© 2025 Cox C, et al. *JAMA Dermatology*.

eTable 3. Participant Cancer Type, Treatment, Treatment Response, and Changes in Clinical AKs From Baseline to 12 Months After Immune Checkpoint Inhibitor Infusion

Cancer Type	Type of ICI	Disease Control at M3	Disease Control at M12	Complete Response at M12	% AK change at M12 or final timepoint	Immune-Related Adverse Events	
						Cutaneous	Other(s)
cSCC	Cemip	Yes	Yes	N/A	-83.4	Nil	Nil
cSCC	Cemip	Yes	Yes	No	-40	Nil	Nil
cSCC	Nivo	Yes	Yes	No	-65.6	Nil	Nil
cSCC	Cemip	Yes	Yes	Yes	-50.6	Rash (BP)	Nil
cSCC	Cemip	Yes	Yes	No	-66.7	Nil	Nil
cSCC	Cemip	Yes	Yes	Yes	-46.4	Nil	Fatigue
Melanoma	Ipi/Nivo	Yes	Yes	Yes	-57.1	Rash	Chronic cough
Melanoma	Nivo	N/A	N/A	*Deceased pre M6*	-30.9	Nil	Nil
Melanoma	Ipi/Nivo	Yes	Yes	Yes	-80.0	Rash	Nil
Melanoma	Ipi/Nivo	Yes	Yes	Yes	-82.1	Rash	Pneumonitis, hepatitis
Melanoma	Nivo	Adjuvant No recurrence		No Recurrence	-61.6	Nil	Nil
Melanoma	Ipi/Nivo	Yes	N/A	No	-66.7	Nil	Colitis, Myasthenia
Melanoma	Nivo	Adjuvant No recurrence		No Recurrence	-77.6	Nil	Nil
Lung	Pembro	N/A	N/A	*Deceased pre M3*	N/A	Nil	Nil
Lung	Ipi/Nivo	Yes	Yes	Yes	-42.9	Rash	Nil
Lung	Ipi/Nivo	Yes	Yes	No	-57.4	Rash	Xerostomia
Lung	Pembro	Yes	Yes	No	-16.7	Nil	Nephritis
Lung	Ipi/Nivo	No	No	*Deceased pre M6*	-69.4	Rash	Thyroiditis, arthralgia, xerostomia
Lung	Pembro	Yes	Yes	No	-75	Nil	Nil
Lung	Pembro	Yes	No	No	-100.0	Nil	Arthralgia
Lung	Ipi/Nivo	No	N/A	*Deceased pre M12*	-53.8	Nil	Nil
Tonsillar	Nivo	N/A	N/A	N/A	-69	Nil	Nil
RCC	Nivo	No	No	No	-81	Nil	Nil

BP = bullous pemphigoid; Cemip = Cemiplimab; ICI = Immune checkpoint inhibitor; Ipi/Nivo = combination Ipilimumab and Nivolumab therapy; M3 = Month 3; M6 = Month 6; M12 = Month 12; N/A = not available or applicable; Nil=no adverse effect; Nivo = Nivolumab; Pembro = Pembrolizumab.

© 2025 Cox C, et al. *JAMA Dermatology*.

eTable 4. The Number of KCs in the Year Before and After Starting ICI Therapy

	Keratinocyte Carcinomas				
	**cSCC**	**BCC**	**IEC**	**KA**	**Total**
**12-months pre-starting ICI therapy**	16	6	19	1	42
**12-months post starting ICI therapy**	5	3	8	1	17

cSCC = Cutaneous Squamous Cell Carcinoma; BCC = Basal cell carcinoma; ICI = immune checkpoint inhibitor; IEC= Intra epidermal carcinoma; KA= Keratoacanthoma.

© 2025 Cox C, et al. *JAMA Dermatology*.
