# Supplementary material for: Immune Checkpoint Inhibitors in Field Cancerization and Keratinocyte Cancer Prevention
Source: JAMA Dermatol. 2025 Feb 12;161(4):383–90. doi: 10.1001/jamadermatol.2024.5750 (PMC11822595; doi:10.1001/jamadermatol.2024.5750)
Supplement: Supplement 2. — Data Sharing Statement [file jamadermatol-e245750-s002.pdf]

Data Sharing Statement

Cox. Association of Immune Checkpoint Inhibitors With Field Cancerization and Keratinocyte Cancer Prevention. *JAMA Dermatol*. Published February 12, 2025.
doi:10.1001/jamadermatol.2024.5750

Data
Data available: Yes
Data types: Deidentified participant data, Data (not involving human participants), Data dictionary
How to access data: Authors are happy to share individual patient data for research purposes as per University of Queensland policy upon request.
When available: With publication

Supporting Documents
Document types: None

Additional Information
Who can access the data: Researchers whose purpose has been approved
Types of analyses: For research purposes
Mechanisms of data availability: With investigator support after ethics approval and signed data access agreement.
